# The exceptional longevity of the naked mole‐rat may be explained by mitochondrial antioxidant defenses

**DOI:** 10.1111/acel.12916

**Published:** 2019-02-15

**Authors:** Daniel Munro, Cécile Baldy, Matthew E. Pamenter, Jason R. Treberg

**Affiliations:** ^1^ Department of Biological Sciences University of Manitoba Winnipeg Manitoba Canada; ^2^ Department of Biology University of Ottawa Ottawa Ontario Canada; ^3^ Centre on Aging University of Manitoba Winnipeg Manitoba Canada; ^4^ University of Ottawa Brain and Mind Research Institute Ottawa Ontario Canada; ^5^ Department of food and Human Nutritional Sciences University of Manitoba Winnipeg Manitoba Canada

**Keywords:** antioxidants, *Heterocephalus glaber*, mitochondria, reactive oxygen species, skeletal muscle heart

## Abstract

Naked mole‐rats (NMRs) are mouse‐sized mammals that exhibit an exceptionally long lifespan (>30 vs. <4 years for mice), and resist aging‐related pathologies such as cardiovascular and pulmonary diseases, cancer, and neurodegeneration. However, the mechanisms underlying this exceptional longevity and disease resistance remain poorly understood. The oxidative stress theory of aging posits that (a) senescence results from the accumulation of oxidative damage inflicted by reactive oxygen species (ROS) of mitochondrial origin, and (b) mitochondria of long‐lived species produce less ROS than do mitochondria of short‐lived species. However, comparative studies over the past 28 years have produced equivocal results supporting this latter prediction. We hypothesized that, rather than differences in ROS generation, the capacity of mitochondria to consume ROS might distinguish long‐lived species from short‐lived species. To test this hypothesis, we compared mitochondrial production and consumption of hydrogen peroxide (H_2_O_2_; as a proxy of overall ROS metabolism) between NMR and mouse skeletal muscle and heart. We found that the two species had comparable rates of mitochondrial H_2_O_2_ generation in both tissues; however, the capacity of mitochondria to consume ROS was markedly greater in NMRs. Specifically, maximal observed consumption rates were approximately two and fivefold greater in NMRs than in mice, for skeletal muscle and heart, respectively. Our results indicate that differences in matrix ROS detoxification capacity between species may contribute to their divergence in lifespan.

## 1 INTRODUCTION

Naked mole‐rats (NMRs; *Heterocephalus glaber*, Rodentia) are mouse‐sized eusocial mammals native to Eastern Africa that live in large subterranean colonies. Individuals of this species can live for >30 years in laboratory conditions (Edrey, Hanes, Pinto, Mele, & Buffenstein, 2011), and also exhibit a remarkably long health span; typical signs of senescence seen in old rodents (e.g., loss of fecundity, lordokyphosis, decreased thermoregulation capacities, elevated incidence of aging‐related disease such as cancer, neurodegeneration, cardiac disorders, muscle atrophy, and increasing mortality rate) are mostly absent in NMRs (Buffenstein, 2008; Edrey et al., 2011; Ruby, Smith, & Buffenstein, 2018). Conversely, the common mouse (*Mus musculus*, Rodentia) lives <4 years and is highly susceptible to aging‐related diseases and physiological decline (Kujoth et al., 2005; Trifunovic et al., 2004). As a result, comparisons between these two species are considered to be a “gold standard” in mammalian studies of aging (Buffenstein, 2005; Dammann, 2017; Edrey et al., 2011).

According to the oxidative stress theory of aging (Barja, 2013; Harman, 1956), senescence is caused by the gradual accumulation of oxidative damage to cells, inflicted by reactive oxygen species (ROS) of mitochondrial origin. However, previous comparative studies of NMR biology mostly provided evidence that contradicted this theory. For example, comparisons of isolated heart mitochondria found no difference in the rate of H_2_O_2_ efflux (i.e., the proportion of H_2_O_2_ not consumed by the mitochondrion before detection, Munro, Banh, Sotiri, Tamanna, & Treberg, 2016) between NMRs and mice (Lambert et al., 2007). In addition, extensive oxidative damage and limited antioxidant capacity have been reported in the cytosol of NMR hepatocytes (Andziak, O'Connor, & Buffenstein, 2005; Andziak et al., 2006). Taken together, these findings led to the conclusion that the longevity of NMRs occurs independently of enhanced protection against oxidative damage (reviewed in Lewis, Andziak, Yang, & Buffenstein, 2013), and this conclusion has been used repeatedly to refute the oxidative stress theory of aging (Hekimi, Lapointe, & Wen, 2011; Robb, Christoff, Maddalena, & Stuart, 2014; Stuart, Maddalena, Merilovich, & Robb, 2014).

More recently, however, the *mitochondrial* oxidative stress hypothesis of aging has gained empirical support (Barja, 2013; Dai, Chiao, Marcinek, Szeto, & Rabinovitch, 2014; Kujoth et al., 2005; Kukat & Trifunovic, 2009; Pamplona, 2011; Shabalina et al., 2017; Trifunovic et al., 2004); however, this hypothesis remains controversial (Stuart et al., 2014), and has not yet been investigated in NMRs. This refined hypothesis stems from the fact that mitochondrial ROS are mostly released inside the mitochondrion (i.e., within the mitochondrial matrix), thereby directly exposing mitochondrial biomolecules to oxidative damage. According to the mitochondrial stress hypothesis, cellular senescence is primarily driven by loss of mitochondrial function with age. A central step toward testing this hypothesis would be to measure the balance between internal production and internal consumption of ROS within mitochondria themselves.

**Figure 1:**
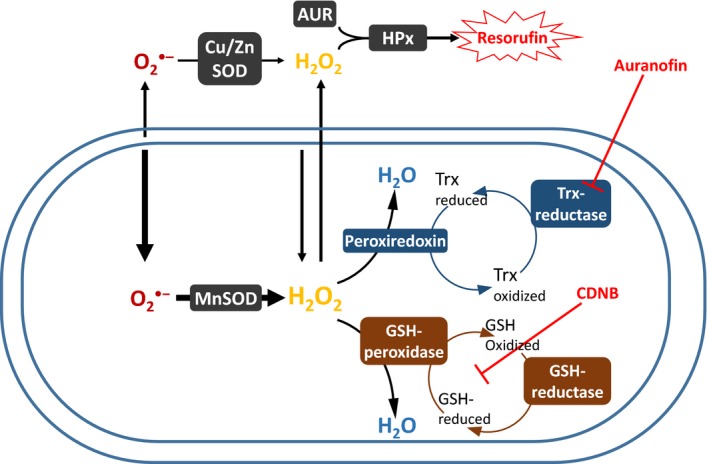
Metabolism of H_2_O_2_ during Horseradish peroxidase‐based efflux assays. Reactive oxygen species (ROS) are generated on either side of the inner membrane, mostly under the form of superoxide (O_2_^•−^) but also directly as H_2_O_2_. Superoxide released inside and outside the matrix will be converted into H_2_O_2_ by the Cu/ZnSOD and MnSOD, respectively. The proportion released inside is additive with the existing pool of H_2_O_2_, leading to two ultimate fates: (a) diffusion across membranes to reach the detection system, or (b) consumption by matrix‐based antioxidants pathways. The reductases of the GSH‐ and Trx‐dependent pathways are activated by provision of NADPH, when substrate is oxidized, and thus concomitantly with ROS formation. Inhibitors for the GSH (CDNB)‐ and Trx (auranofin)‐dependent pathways (also used in this study) are depicted in red

We have recently shown that traditional methodologies for detecting the rate of H_2_O_2_ formation from isolated mitochondria underestimate ROS generation because of the remarkable endogenous capacity of matrix antioxidants to consume H_2_O_2_. For example, this underestimation can reach >80% in rat skeletal muscle with certain respiratory substrates (See Figure 1 in methods; Munro et al., 2016). Moreover, mitochondria can consume far more H_2_O_2_ than they generate (Drechsel & Patel, 2010; Starkov et al., 2014; Zoccarato, Cavallini, & Alexandre, 2004); therefore, this capacity of mitochondria to consume H_2_O_2_ putatively represents a novel and widely underappreciated test of the *mitochondrial* oxidative stress theory of aging in of itself. We hypothesized that differences in the capacity of mitochondria to eliminate H_2_O_2_ might solve the apparent NMR oxidative stress/longevity‐conundrum (Lewis et al., 2013).

To test our hypothesis, we took advantage of antioxidant inhibition methods that we developed previously (Munro et al., 2016) to measure H_2_O_2_ formation rates without the confounding influence of internal consumption (Figure 1). We also compared mitochondrial H_2_O_2_ clearance (i.e., maximal consumption) rates between these two species in functional isolated mitochondria (Drechsel & Patel, 2010; Lopert & Patel, 2014; Munro et al., 2016; Starkov et al., 2014; Zoccarato et al., 2004). Our results support the *mitochondrial* oxidative stress hypothesis of aging via a mechanism that has not been previously demonstrated: NMRs and mice do not differ in their rate of H_2_O_2_ formation, but rather in the markedly greater capacity of NMR mitochondria to consume H_2_O_2_.

## 2 RESULTS

### 2.1 Oxygen consumption

Mitochondrial oxygen consumption was measured simultaneously with H_2_O_2_ formation, and these respiration rate data are reported in the Supporting Information (Figures S1 and S2). When measured at the species’ respective body temperatures, the respiratory control ratio (RCR) values for NMR and mouse skeletal muscle mitochondria, respectively, were (mean ± *SEM*) 11.0 ± 0.4 and 9.8 ± 0.6 when fueled by malate + glutamate, and 3.7 ± 0.4 and 2.8 ± 0.1 when fueled by succinate + rotenone. These values were not significantly different between species within each substrate condition (Supporting Information Figure S2). Using malate + glutamate, the RCR of heart mitochondria was significantly higher for NMRs 21.0 ± 0.7 compared to mice 7.6 ± 0.4 (*p* < 0.0001). Our mouse heart mitochondria nonetheless had a higher RCR for NADH‐generating substrates than reported by other groups (range = 3–6; Graham et al., 1997; Hughes & Hekimi, 2011).

### 2.2 The rate of H_2_O_2_ formation is not consistently lower in NMR muscle or heart mitochondria

We recently validated pharmaceutical inhibition methods that compromise the glutathione (GSH)‐ and thioredoxin (Trx)‐dependent ROS scavenging pathways in skeletal muscle mitochondria of rodents, without affecting mitochondrial energetics (Figure 1; Munro et al., 2016). By blocking the endogenous consumption of H_2_O_2_ by mitochondria, this inhibitory approach allows us to accurately estimate the combined rate of superoxide and H_2_O_2_ formation using fluorescence assays based on mitochondrial H_2_O_2_ efflux.

**Figure 2:**
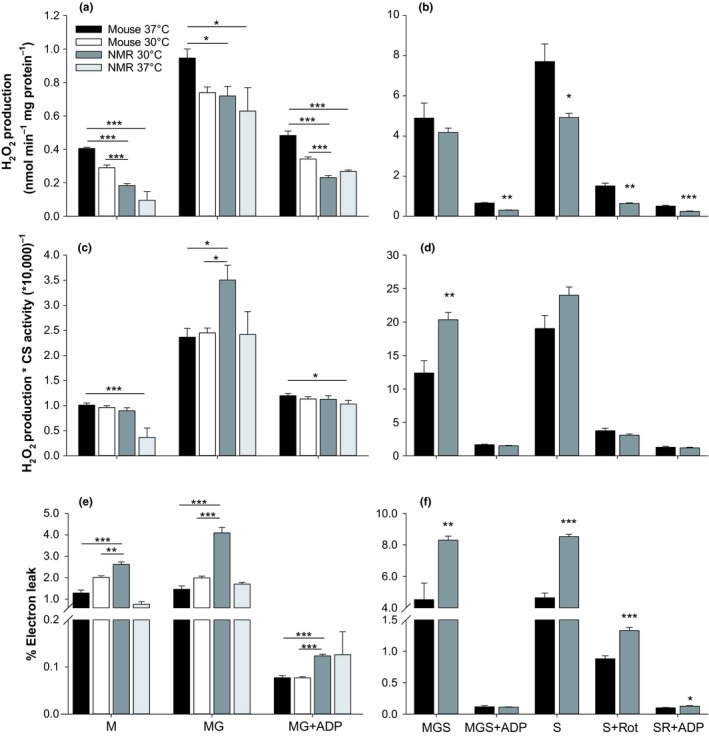
Rates of H_2_O_2_ production by isolated muscle mitochondria of NMRs and mice. The two respiration‐dependent pathways for the matrix consumption of H_2_O_2 _(see Figure 1) were inhibited by CDNB pre‐treatment and auranofin. (a, c, and e) assays for both species at the normal body temperature of the naked mole‐rat (NMR; 30°C) and the mouse (37°C). (b, d, and f), results are limited to assaying each mitochondrial preparation at each species’ normal body temperature. Data are normalized to mg of mitochondrial protein (a and b), to the activity of the citrate synthase measured at the same temperature (c and d), or as the % of electrons leaking from the electron transfer system toward the formation of H_2_O_2_ (e and f). M: malate; G: glutamate; Rot: rotenone; S: succinate. Significant differences between species were assessed using *t* tests, with *p < 0.05, **p < 0.01, and ***p < 0.001. For mouse, *n* = 4 for M and MG, *n* = 9 for MGS and MGS + ADP, and *n* = 7 for all conditions with succinate. For NMR, *n* = 6 for all conditions except at 37°C (*n* = 2). Data are mean ± *SEM*, except for NMR at 37°C where bars represent range

When normalized to mg of protein, H_2_O_2_ production rates from skeletal muscle mitochondria were higher for the mouse than for the NMR, with the exception of experiments in which malate + glutamate + succinate were supplied simultaneously in the absence of ADP (Figure 2a,b). The differences between the two species tended to be greater in conditions in which H_2_O_2_ is produced at low, and therefore, more physiologically relevant rates. When normalized to the activity of the mitochondrial‐specific enzyme citrate synthase, rates of H_2_O_2_ production did not show any consistent trend between species and across conditions, with rates being higher for the NMR with malate + glutamate and for malate + glutamate + succinate (Figure 2c,d), but lower with malate or malate + glutamate + ADP when mitochondria of both species are measured at 37°C. The production of H_2_O_2_ can also be expressed as a proportion of electrons prematurely “leaking” from the electron transport system for the single‐electron reduction of oxygen (O_2_) into superoxide (O2^•−^) or the two‐electron reduction of oxygen to H_2_O_2_. The % electron leak was consistently higher from NMR mitochondria at 30°C than from mouse mitochondria at either 30 or 37°C across assay conditions, with the exception of malate + glutamate + succinate in the presence of ADP (Figure 2e,f). Therefore, depending on the denominator chosen, completely different conclusions regarding which species produces more or less H_2_O_2_ may be supported by the same dataset.

**Figure 3:**
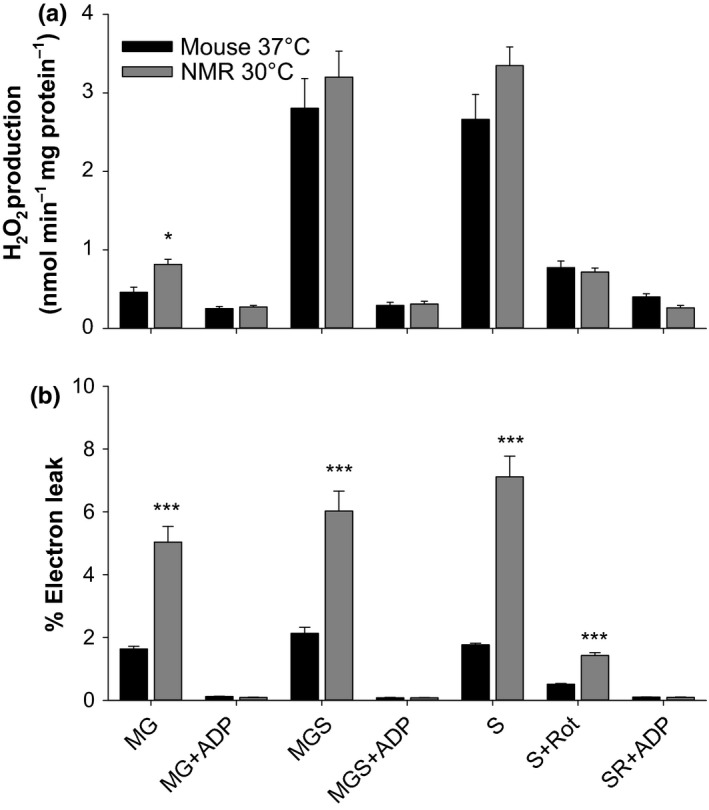
Rate of H_2_O_2_ production from isolated heart mitochondria of the NMR and the mouse. Data are normalized to mg of mitochondrial protein (a), or as the % of electrons leaking from the electron transfer system toward the formation of H_2_O_2_ (b). Sample sizes are *n* = 3 and *n* = 5 for NMR and mice, respectively. Each mitochondrial preparation was obtained by pooling four hearts. Other details and conditions of assay are as in Figure 2

To determine whether these differences persisted in other tissues, we also measured the production of H_2_O_2_ from heart mitochondria at each species’ physiological temperatures and normalized results to mg of protein. We found no significant differences between species except for a higher production of H_2_O_2_ in NMR mitochondria during malate + glutamate oxidation (Figure 3a). Electron leakage was found to be much higher from NMR mitochondria than from mouse mitochondria in the absence of ADP, however, when ADP was present, this difference vanished, with a trend toward lower % leakage in NMR for malate + glutamate + ADP (Figure 3b; *p* = 0.079).

### 2.3 H_2_O_2_ consumption is consistently higher in NMR mitochondria

**Figure 4:**
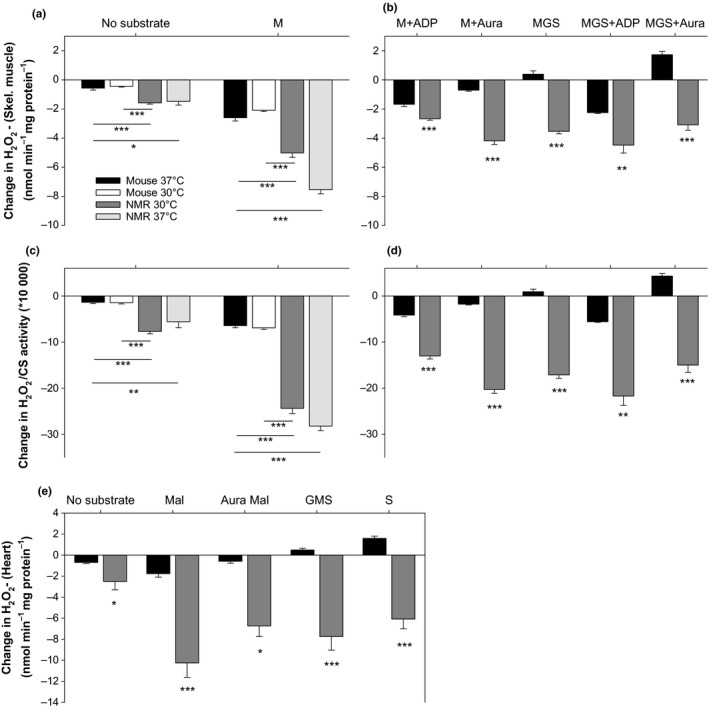
Consumption of H_2_O_2_ by untreated skeletal muscle (a–d), and heart (e) mitochondria after exogenous addition of 2.5 µM H_2_O_2_. Negative values represent net consumption, positive values represent net production (see Munro et al., 2016 for methodological details). Aura = auranofin, an inhibitor of the thioredoxin reductase. For mouse skeletal muscle, *n* = 4 for all conditions, except for no substrate and M at 37°C, where *n* = 6. For NMR skeletal muscle at 37°C, *n* = 3; at 30°C, *n* = 4 for MGS +ADP, *n* = 5 for MGS and MGS + Aura, and *n* = 6 for all other conditions. For the heart, *n* = 4 (4 animals per preparation) for all. Other details are as in Figure 2

The capacity of matrix consumers of H_2_O_2_ (Figure 1) can be assessed by challenging isolated mitochondria with a bolus of H_2_O_2_ and following the rate at which [H_2_O_2_] decays (Drechsel & Patel, 2010; Starkov et al., 2014; Zoccarato et al., 2004). In our experiments, negative values indicate that H_2_O_2_ consumption is occurring faster than H_2_O_2_ formation. Previous work with rat skeletal muscle showed that malate alone is sufficient to spark full activity of the respiration‐dependent GSH and Trx pathways, while simultaneously minimizing the H_2_O_2_ formation rate (Treberg, Munro, Banh, Zacharias, & Sotiri, 2015, and Figures 1 and 4). Rates of H_2_O_2_ consumption with malate therefore provide an appropriate estimation of maximal H_2_O_2_ consumption in the presence of 2.5 µM H_2_O_2_ (Treberg et al., 2015).

For skeletal muscle, the maximal rate of H_2_O_2_ consumption with malate was much higher for NMR than for mouse mitochondria, irrespective of normalization to mg of protein or to the activity of citrate synthase (Figure 4a,c). Moreover, the excess H_2_O_2_ consumption capacity (relative to production) of NMR muscle mitochondria extended to all combinations of substrates and effectors tested (which concomitantly produce H_2_O_2_ to varying degrees), again regardless of normalization to mg of protein or citrate synthase (Figure 4b,d). These conditions also included the residual consumption of H_2_O_2_ in the absence of exogenously added respiratory substrates (Figure 4a,c). Note that positive values for the change in [H_2_O_2_], indicating net formation, were only seen in mice mitochondria when succinate was present and in absence of ADP (Figure 4b,d).

For heart mitochondria, the maximal rate of H_2_O_2_ consumption, estimated with malate, was more than five times greater for the NMR than for the mouse (Figure 4e). Again, significantly higher rates of consumption were found for the NMR in all conditions tested, when simultaneous production of H_2_O_2_ is above negligible, with positive values only found for the mouse (Figure 4e).

**Figure 5:**
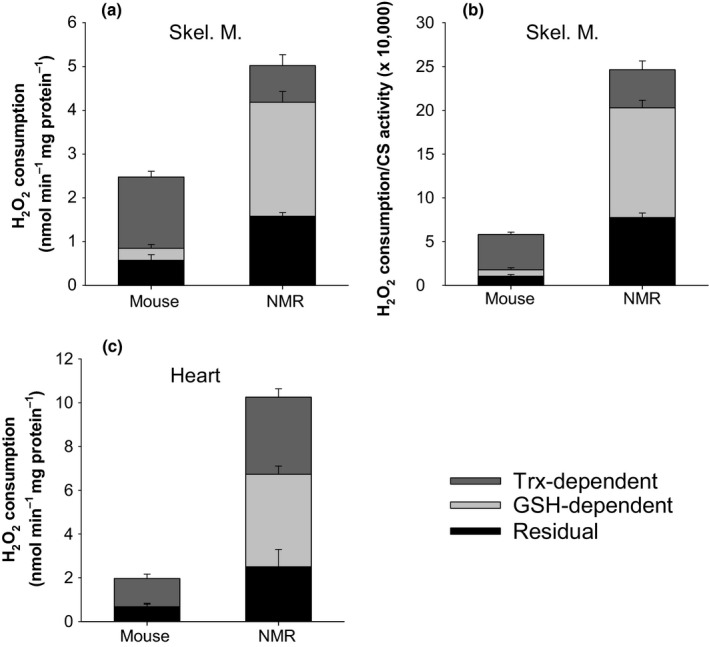
Cumulative allocation of H_2_O_2_ consumption among the three major matrix consumers of H_2_O_2_ for skeletal muscle (a and b) and heart (c) mitochondria. Total consumption corresponds to the maximal consumption obtained during malate oxidation (from Figure 4, see text for rationale). The Trx‐dependent proportion was defined by subtracting consumption with malate + auranofin from total consumption (See Figure 1). Residual consumption is the consumption in absence of exogenously added substrate. The GSH‐dependent proportion was defined as the proportion of total consumption not explained by the sum of the Trx‐dependent pathway and residual consumption, on the premise that only the three consumers of H_2_O_2_ considered here are important in the matrix. Residual consumption can be explained by the activity of catalase as well as by residual activity of the respiration‐dependent pathways for the consumption of H_2_O_2_, which are partially energized by endogenous (slow oxidizing) substrates remaining in the matrix after mitochondrial isolation (see Munro et al., 2016). Catalase was previously found to represent a negligible component of the residual consumption for rat skeletal muscle mitochondria (Munro et al., 2016), but the higher levels of residual consumption found here for the NMR in both tissues suggests at least some involvement of catalase

When auranofin is used to inhibit the Trx‐dependent pathway during H_2_O_2 _consumption assays, the remaining consumption can be attributed to the GSH‐dependent pathway and the respiration‐independent residual consumption. Residual consumption is quantified by simply omitting substrate. Subtracting each source of H_2_O_2_ consumption from total consumption resolves the partitioning of the three sources of matrix H_2_O_2_ consumption in each species. Figure 5a,b shows that, for NMR, the GSH‐dependent pathway predominates over the Trx‐dependent pathway in muscle mitochondria, whereas the Trx‐dependent pathway predominates in mouse. For the heart, both pathways are approximately equally active in NMR mitochondria whereas the Trx‐dependent pathways still predominate in the mouse (Figure 5c).

### 2.4 Normalizing H_2_O_2_ production to the rate of consumption

**Figure 6:**
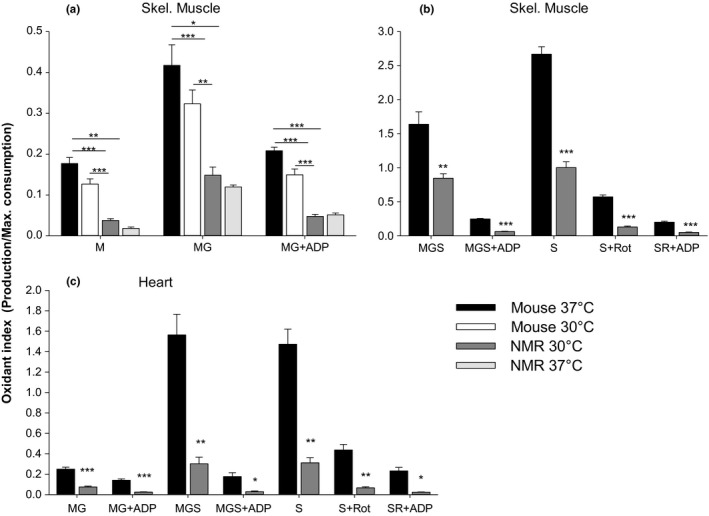
Oxidant index (H_2_O_2_ production rate/maximal H_2_O_2_ consumption rate) for skeletal muscle (a and b) and heart (c) mitochondria. See text for the rationale of normalizing to maximal rate of H_2_O_2_ consumption. Data are from Figures 2, 3, 4. Details and statistics are as in Figure 2 (skeletal muscle) and Figure 3 (heart)

The degree of oxidative insult sustained by mitochondria ultimately depends on the balance between ROS formation and elimination within the organelle. Our approach of separating H_2_O_2_ formation rates from H_2_O_2_ consumption rates allowed us to integrate our data for the purpose of comparing the potential for self‐inflicted oxidative damage between species. Formation rates, for each respiratory substrate condition, were divided by maximal consumption rates, which is likely invariable across substrate conditions used in the current study (Munro & Treberg, 2017; Treberg et al., 2015), providing an oxidant index. Figure 6a,b shows that the oxidant index of skeletal muscle mitochondria is substantially lower for NMRs in all conditions of temperature or substrate tested. In particular, note the pronounced difference between the two species in the more biologically relevant conditions where: (a) ATP is being generated (i.e., in the presence of ADP), and (b) the electron transport system is fed mainly through complex I (malate and glutamate) or both complex I and II simultaneously (malate + glutamate + succinate). Figure 6c shows that the oxidant index difference is even larger for heart mitochondria, being up to 6.1 times lower for the NMR in the physiologically relevant condition of MGS + ADP.

### 2.5 Relative rates of activity among complexes of the electron transport system

**Figure 7:**
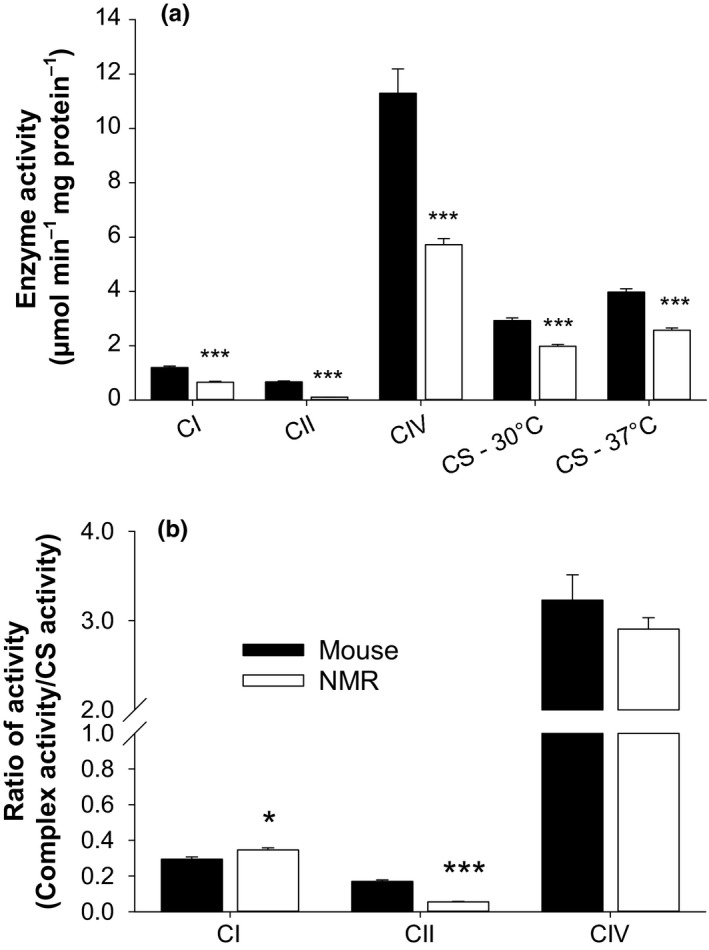
Activity of electron carrying complexes, and the Kreb's cycle enzyme citrate synthase. Data were acquired using untreated skeletal muscle mitochondria thawed only once, except for complex I activity which requires three cycles of freeze‐thaw (Spinazzi et al., 2011). Assays were conducted at the species’ normal body temperature and normalized to mg of protein (a), or to the activity of citrate synthase (b). CI = complex I, CII = complex II, CIV = complex IV, CS = citrate synthase, NMR = naked mole‐rat. In panel a, for mouse, *n* = 15, 16, 15, 10, 11 from left to right, for NMR, *n* = 8 except for CI, where *n* = 6. In panel b, for mouse *n* = 10 (except for CI, *n* = 11), for NMR, *n* = 8 (except for CI, *n* = 6). Other details of statistics are as in Figure 2

Past studies often attributed the lower rate of H_2_O_2_ efflux observed in mitochondria of long‐lived species (during succinate oxidation) to a lower content (or activity) of complex I of the electron transport system (Ayala et al., 2007; St‐Pierre, Buckingham, Roebuck, & Brand, 2002). We measured enzymatic activity of complexes I, II, and IV in skeletal muscle mitochondria to test this possibility. Figure 7 shows the activity of complexes of the electron transport system measured at each species’ normal body temperature. As predicted by the hypothesis, the activity of complex I is lower for the NMR than for the mouse when normalized to mg of protein, but the same is found for the activity of all other complexes as well as for citrate synthase (Figure 7a). This finding could simply suggest that NMRs possess a lower content of enzymes involved in oxidative phosphorylation relative to overall mitochondrial protein. Indeed, when normalized to the activity of citrate synthase, complex I activity was higher in NMRs compared to murine mitochondria, whereas complex IV activity was no longer different between species (Figure 7b). A more consistent difference between species, however, is the remarkably lower activity of complex II in NMR muscle, irrespective of the marker used for normalization.

## 3 DISCUSSION

Mitochondrial ROS have long been suspected to have a direct role in cellular senescence. Comparative studies have been conducted across three decades that attempted to correlate mitochondrial H_2_O_2_ efflux (which were assumed to represent endogenous rates of H_2_O_2_ formation) with animal longevity (Barja, 2013), without providing a clear answer (Lambert et al., 2007; Lambert & Brand, 2007). Here, we take a radically different approach: we use methods that aim to eliminate internal consumption of H_2_O_2_ during measurements of H_2_O_2_ efflux from isolated mitochondria (Munro et al., 2016; Treberg et al., 2015), thereby providing an accurate estimation of mitochondrial superoxide and H_2_O_2_ production. We also assess the capacity of isolated mitochondria to consume external H_2_O_2_ as a more relevant test of their integrated capacities for H_2_O_2_ detoxification in vivo. The results are surprising, we do not find a clear difference between species regarding the rate of H_2_O_2_ formation in this (strong) comparative model; instead, we report a markedly greater capacity of NMR mitochondria to detoxify H_2_O_2_.

### 3.1 Matrix antioxidants and aging

Antioxidant concentrations and activities do not generally correlate (negatively or positively) with longevity (reviewed in Hulbert, Pamplona, Buffenstein, & Buttemer, 2007); however, these investigations rarely focussed on mitochondrial antioxidants despite early evidence that the relationship may work better in this case. For example, looking across three age classes, it is not obvious that the activity of the cytosolic (and intermembrane space)‐localized superoxide dismutase (Cu/ZnSOD), and total catalase activity are higher for the NMRs as compared to mice, and the cytosolic glutathione peroxidase is clearly more active in mice than in NMRs. However, the matrix‐specific superoxide dismutase (MnSOD) is more active in NMRs than in mice for the young‐ and middle‐aged classes (Andziak et al., 2005).

Importantly however, measuring the isolated activity of matrix‐specific peroxidases or reductases is likely insufficient to draw meaningful conclusions in comparative studies of aging. Several lines of evidence support this conclusion. First, our study and previous studies using intact mitochondria (Figure 4; Drechsel & Patel, 2010; Starkov et al., 2014) show that the relative contribution of each of the GSH‐ and Trx‐dependent pathways is highly variable across species and tissues. Therefore, targeting one or the other of the peroxidases linked to these pathways (i.e., the GSH peroxidase or the peroxiredoxine) may be misleading. Second, in vivo, matrix antioxidant enzymes work as part of an integrated pathway (i.e., the GSH‐ or Trx‐dependent pathways) responsible for providing reducing equivalents to the peroxidase (Figure 1). To our knowledge, very little is known regarding the relative contribution to overall flux control exerted at each steps of these pathways. Indeed, this rationale can be extended to the whole mitochondrion—that is, the reductases for the GSH‐ and Trx‐dependent pathways are exclusively dependent on the provisioning of NADPH (Drechsel & Patel, 2010; Munro et al., 2016; Starkov et al., 2014; Treberg et al., 2015; Zoccarato et al., 2004), originating from the oxidation of respiratory substrates by the Krebs cycle and directly from other matrix enzymes. Third, it has been suggested that a protonmotive force across the inner mitochondrial membrane is necessary for the conversion of NADH to NADPH in certain species (Lopert & Patel, 2014). Too many influential aspects of the complete metabolism of ROS thus seem to be ignored when the maximal activity of isolated antioxidant enzymes is used to permit inferences to the mitochondrion as a whole. Conversely, our approach of adding a bolus of H_2_O_2_ to the respiration medium overcomes these limitations very simply and directly since H_2_O_2_ can cross both membranes and interact directly and equally with all antioxidants of the matrix within a functional and energized mitochondria.

In addition to the difficulties in comparisons across species, matrix antioxidants may have also been overlooked with respect to their absolute importance in establishing ROS balance in the mitochondrion. The maximal H_2_O_2_ consumption capacities of intact mitochondria are generally far in excess of their maximal capacities for H_2_O_2_ formation in the absence of respiratory inhibitors (Drechsel & Patel, 2010; Munro et al., 2016; Starkov et al., 2014; Zoccarato et al., 2004). Changes in the scale of activity across species and tissue also seem more important than what is generally found for ROS formation (Figure 3, Lambert et al., 2007). Here, we found that, for heart mitochondria, the maximal capacity for the consumption of H_2_O_2_ was more than five times higher for the NMR as compared to mice, pointing to a major means of modulating the overall H_2_O_2_ balance (Munro & Treberg, 2017).

Previous genetic and pharmaceutical interventions have already suggested a potential link between mitochondrial detoxification of H_2_O_2_ and lifespan in other models. For example, the overexpression of catalase targeted to mitochondria is one of the rare genetic interventions that can extend the lifespan of vertebrates (Dai et al., 2009; Schriner et al., 2005). Similarly, synthetic antioxidants chemically targeted to mitochondria (e.g., SkQ1) can extend healthspan of wild‐type rodents (Skulachev et al., 2009), and both healthspan and lifespan of mtDNA mutator mice (Shabalina et al., 2017). Furthermore, the (genetically engineered) loss of the antioxidant and electron carrier ubiquinone results in the partial loss of mitochondrial function and shortened lifespan in a mouse model (Wang, Oxer, & Hekimi, 2015).

### 3.2 Traditional measures of mitochondrial H_2_O_2_ production should be interpreted with caution

Previous studies measured the proportion of H_2_O_2_ diffusing out of the mitochondrion, which represent the proportion not consumed by matrix antioxidants. The problem with this approach is that most superoxide (and thus ultimately most H_2_O_2_) is produced inside the mitochondrion, and as much as 80% may be consumed therein. As a result, this contribution remains “invisible” to the detection system. Therefore, differences reported between species using such a detection system may equally result from mitochondria having a lower H_2_O_2_ formation rate per se as from mitochondria having greater H_2_O_2_ consumption capacities (Munro et al., 2016; Treberg et al., 2015; Treberg, Quinlan, & Brand, 2010).

A particularly problematic aspect of the internal consumption of H_2_O_2_ with traditional measures is that the lower the *actual* rate of H_2_O_2_ formation, the greater the proportion that is consumed inside mitochondria (Munro et al., 2016). Unfortunately, experimental conditions using substrates that are generally considered more representative of the in vivo milieu (e.g., in presence of ADP and a complex assemblage of respiratory substrates at typically sub‐saturating concentrations) generally tend to produce less H_2_O_2_ (Goncalves, Quinlan, Perevoshchikova, Hey‐Mogensen, & Brand, 2015). Hence, it is very likely that the more representative a combination of substrate and effectors is of the in vivo milieu, the greater the difference between species actually (albeit indirectly) represents a difference in matrix H_2_O_2_ consumption capacities.

### 3.3 The choice of denominator impacts conclusions

Previous comparative studies have mostly used protein content in the mitochondrial pellet as a means of normalizing rates of H_2_O_2_ formation by mitochondria. Normalizing H_2_O_2_ formation to the consumption of oxygen in the same condition of respiratory substrates is also often used. Here, we tested these two markers of mitochondrial density when normalizing H_2_O_2_ formation rate. In addition, we also used the activity of citrate synthase. These three denominators provided us with three different conclusions with respect to determining which species produces more H_2_O_2_ (Figure 2). The degree of confusion, introduced by the choice of denominator, is therefore not acceptable if a conclusion is to be reached concerning a possible relationship between longevity and the rate of H_2_O_2_ formation across species.

### 3.4 The oxidant index as an unbiased measure of oxidative insult inside mitochondria

In this study, we divided the rate of H_2_O_2_ formation by the maximal rate of H_2_O_2_ consumption to obtain an oxidant index. This approach avoids any bias associated with the use of a particular marker of mitochondrial density. For instance, the oxidant index is not biased by changes in the density of electron transport system enzyme complexes, the capacity for oxygen consumption, or the protein content of mitochondria across species. Instead, the oxidant index directly assesses the ratio between the opposing processes of production and elimination of H_2_O_2_ within the matrix, and thereby allows the direct determination of whether or not the matrix of one species sustains a higher degree of oxidative insult. We suggest that the oxidant index has the potential to eliminate many confounding factors in the field that have precluded the reaching of a consensus conclusion regarding a putative relationship between longevity and mitochondrial ROS in previous studies (Lambert et al., 2007; Lambert & Brand, 2007; Montgomery, Hulbert, & Buttemer, 2012).

### 3.5 Does the NMR biogerontology model support a role for oxidative damage in aging?

Results from the comparison between the NMR and the mouse have often been presented as evidence against the hypothesis that oxidative damage is involved in aging and longevity, and in particular (curiously) to refute the now obsolete oxidative stress theory of aging (Hekimi et al., 2011; Lewis et al., 2013; Stuart et al., 2014). For example, higher steady‐state levels of oxidative stress markers including protein carbonyls, urinary and cellular isoprostanes, liver 8‐OHdG, malondialdehyde, and a lower activity of cytosolic glutathione peroxidase have been found in NMR hepatocytes compared to murine hepatocytes (Andziak et al., 2005, 2006). Many authors however do not distinguish between the *classical* oxidative stress theory of aging, first suggested by Harman in 1956 (i.e., that senescence is the result of oxidative damage to cells), and the more recent *mitochondrial* oxidative stress hypothesis of aging (i.e., that senescence is the result of oxidative damage to mitochondria themselves). Whereas the latter hypothesis has received support from transgenic models (Kujoth et al., 2005; Kukat & Trifunovic, 2009; Trifunovic et al., 2004), the former has been convincingly refuted (Hekimi et al., 2011; Stuart et al., 2014). Our finding of better protection against H_2_O_2_ in the matrix of NMR mitochondria, as well as the previous finding of greater protection against superoxide (higher activity of the MnSOD, Andziak et al., 2005), supports the mitochondrial oxidative stress hypothesis of aging. A surprising finding, however, is that recruiting matrix antioxidants rather than downregulating basal ROS production inside the matrix of long‐lived species appears to be the predominant mechanism of protection.

### 3.6 Is there a longevity signature in the stoichiometry of the electron transport system?

Previous studies reported lower activity/content of complex I of the electron transport system and suggested this may represent a mechanism for increasing longevity by decreasing mitochondrial H_2_O_2_ formation rate during “reverse electron transport” (Ayala et al., 2007; St‐Pierre et al., 2002), with succinate as a substrate. Here, we report that complex II, and not complex I, has a distinctively lower activity in the NMR as compared to the mouse (Figure 7). The same trend is found for mantle mitochondria from the longest‐lived metazoan, the Atlantic clam *Arctica islandica*, as compared to shorter‐lived counterparts (i.e., *Mya arenaria* and *Spisula solidissima*, Munro, Pichaud, Paquin, Kemeid, & Blier 2013, Aging Cell). Complex II could be pertinent to mitochondrial oxidant burden in two ways; it can be an important source of superoxide or H_2_O_2_ by itself (Quinlan et al., 2012), and complex II also supplies most of the electrons to the ubiquinone pool during succinate oxidation and the associated “reverse electron flow”. Reverse electron flow is particularly sensitive to the protonmotive force, and higher level of complex II activity would maintain a higher protonmotive force for an equal content of complex I, leading to a higher ROS production rate (Lambert & Brand, 2004). Further studies are required to clarify the pattern of change in mitochondrial electron transport system between short‐ and long‐lived species.

## 4 CONCLUSION

Our finding of increased capacity for H_2_O_2_ consumption in NMR mitochondria has multiple implications. First, it reconciles the biology of the NMR with the *mitochondrial* oxidative stress hypothesis of aging: macromolecules of the matrix suffer lesser basal oxidant insult in long‐lived NMRs than in short‐lived mice. Second, it offers a potential explanation for the lack of consistency across previous comparative studies of aging. If long‐lived species mostly differ from short‐lived ones for enhanced matrix antioxidants capacities, then past studies using traditional measures of H_2_O_2_ efflux might have struggled to identify an inverse relationship with longevity simply because H_2_O_2_ efflux is a poor (and indirect) means of estimating matrix antioxidants. Third, the possibility that the evolution of long lifespan proceeds through upregulation of matrix antioxidants and not by modifying sites of ROS production has profound implications for the medical domain. For instance, such a finding may foster additional interest in developing synthetic antioxidants targeted to mitochondria for the postponement of aging‐related diseases (Shabalina et al., 2017; Skulachev et al., 2009). Future studies are required at this point to investigate whether greater mitochondrial capacity to consume H_2_O_2_ is a generalized trait across long‐lived species relative to their shorter‐lived counterparts, which would represent a major paradigm shift in the field of aging.

## 5 EXPERIMENTAL PROCEDURES

### 5.1 Reagents

Auranofin was obtain from Enzo Life Sciences. CDNB (1‐chloro‐2,4‐dinitro‐benzene) was obtained from Milipore‐SIGMA, Oakville, Canada. Amplex Ultrared was obtained from THERMO‐FISHER, Waltham, MA. All other chemicals and enzymes were purchased from Milipore‐SIGMA or ACROS, Geel, Belgium.

### 5.2 Animals and preparation of isolated mitochondria

All housing conditions and protocols were approved by the local animal care committees of the University of Ottawa and the University of Manitoba, based on the guidelines of the Canadian Council on Animal Care. Male C57BL/6N mice between 10–12 weeks old (20–26 g) were either obtained from the central facility at the University of Manitoba and housed in the Biological Sciences animal care facility (Fort Garry Campus, University of Manitoba; for skeletal muscle data), or obtained through (Charles River), and housed at, the animal care facility of the University of Ottawa (Main campus; heart data). Individuals were fed a standard chow diet ad libitum and sacrificed by CO_2_ asphyxiation followed by rapid cervical dislocation. Male naked mole‐rats of approximately corresponding biological age (i.e., between 2 and 3 years old, 38–50 g) were obtained from the colony of Dr. Matthew Pamenter, University of Ottawa, Canada. Individuals were group‐housed in interconnected cages simulating burrows with tunnel systems, and kept at an ambient temperature of 30°C, and 50% humidity. NMR were fed fresh fruits, vegetables, and Pronutro cereal supplements ad libitum and sacrificed by exposure to isoflurane (skeletal muscle data) followed by cervical dislocation, or directly by cervical dislocation (heart data). For both species, hind legs and dorsal skeletal muscles were dissected and mitochondria were isolated by differential centrifugation as previously described (Affourtit, Quinlan, & Brand, 2012). Mitochondrial protein concentration was determined by the Biuret assay in the presence of 0.2% sodium deoxycholate using bovine serum albumin as a standard.

### 5.3 Inhibition of matrix H_2_O_2_ consumption

Measures of H_2_O_2_ production rates were acquired from mitochondria treated to inhibit matrix‐based H_2_O_2 _consumption (Figure 1). Specifically, the glutathione (GSH)‐dependent enzymatic pathway was inhibited by use of the GSH *S*‐transferase substrate CDNB (1‐chloro‐2,4‐dinitro‐benzene) which irreversibly binds to GSH, as explained in (Treberg et al., 2010). In accordance with preliminary tests for this study, the exposure period of mitochondria to CDNB at room temperature was reduced from 5 to 4 min which prevented changes in respiration rates in the presence of glutamate—malate—succinate and ADP. The Trx‐dependent enzymatic pathway was inhibited by 0.5 µM auranofin, an inhibitor of the thioredoxin reductase, which was added directly to the assay medium. In the absence of calcium (Rigobello, Scutari, Boscolo, & Bindoli, 2002), auranofin has no side effects on mitochondrial energetics, up to a concentration of 2 µM in rats (Munro et al., 2016) and NMRs, and up to 0.5 µM in mice (preliminary tests, present study). Further details of these methods and their degree of selectivity can be found in (Munro et al., 2016; Treberg et al., 2010).

### 5.4 Mitochondrial respiration and H_2_O_2_ production

Rates of oxygen consumption and H_2_O_2_ production were monitored simultaneously using an Oroboros O2K (Oroboros, Innsbruck, Austria) equipped with a fluorescence detection module mounted with the appropriate excitation and emission filters for the fluorescent probe Amplex UltraRed (AUR). Respiration medium consisted of 120 mM KCl, 20 mM Hepes, 5 mM KH_2_PO_4_, 2.5 mM MgCl_2_, 1 mM EGTA, and 0.3% BSA (pH 7.2 at 37°C). Amplex Ultrared (10 µM), horseradish peroxidase (5 IU/ml), superoxide dismutase (25 IU/ml), hexokinase (5 IU/ml), glucose (20 mM), and auranofin (0.5 µM) were added to the respiration medium separately for each assay. Mitochondria (skeletal muscle: 0.12–0.20 and 0.08–0.15, and heart: 0.03–0.05 and 0.05–0.08 mg protein/ml for the NMR and the mice, respectively) were then added to 2 ml of respiration medium (which was constantly mixed via magnetic stirring) that had been air equilibrated at 30°C or 37°C in the Oroboros chamber. The respiration chamber was then closed and appropriate effectors (5 mM malate, glutamate, succinate; 4 µM rotenone; 0.5 mM ADP) were added to initiate the different energetic conditions for each of the three type of sequential assays (SUIT protocols) defined by the substrate combination (i.e., malate + glutamate, malate + glutamate + succinate, or succinate). Baseline values for oxygen consumption and H_2_O_2_ production were measured following addition of each substrate/inhibitor. Rates of fluorescent product (resorufin) formation were converted to nmoles of H_2_O_2_ based on a two‐point standard curve conducted at the end of each assay by direct addition of H_2_O_2 _in the chamber, thereby accounting for chemical interference and fluorescence quenching by mitochondria.

### 5.5 Consumption of H_2_O_2 _(presented as change in H_2_O_2_·min^−1^ in the figures)

Hydrogen peroxide consumption assays were performed with untreated mitochondria as described in (Munro et al., 2016), using between 0.05 and 0.07 (skeletal muscle); and between 0.03 and 0.04 (heart) mg mitochondrial protein.

### 5.6 Enzyme assays (skeletal muscle)

Frozen (−80°C) mitochondria were thawed only once prior to measurements, except for the complex I (NADH‐ubiquinone oxidoreductase) assay which requires three cycles of freeze‐thaw to maximize activity according to (Spinazzi et al., 2011). Assays were conducted at 30 or 37°C (see figures), following the methodology developed by (Spinazzi et al., 2011). For complex IV (cytochrome *c* oxidase) activity, lauryl maltoside was used at 0.05% in order to maximize activity.

### 5.7 Presentation of data

For data acquisition from heart mitochondria, animals had to be pooled by groups of 4 in each species. Due to limited availability of NMRs, we were unable to recover sufficient mitochondria to perform some of the additional assays carried out with skeletal muscle mitochondria. The means of normalizing mitochondrial rates is a matter of debate in comparative studies (Hulbert, Turner, Hinde, Else, & Guderley, 2006; Larsen et al., 2012). With the more abundant skeletal muscle mitochondria, we used a number of frequently used markers of mitochondrial density (mg of mitochondrial protein, citrate synthase activity, and oxygen consumption) to investigate whether differences between species are dependent on the use of one particular denominator or if they represent generalized trends across denominators.

The mesothermic NMR maintains its body temperature at 1–2°C above ambient temperatures when within its thermoneutral zone of 30–34°C (Buffenstein, Park, Hanes, & Artwohl, 2012). NMR colonies were housed at 28–30°C, and therefore we chose 30°C as the assay temperature at which to perform assays examining NMR mitochondria. In contrast, mice have a more typical mammalian body temperature and therefore we performed all measurements in this species at 37°C. With skeletal muscle mitochondria, we also performed several assays at both of these two temperatures (30 and 37°C) in each species. Data collected at only one temperature were measured at the respective physiological temperatures of each species to provide physiologically relevant comparisons (NMR at 30°C and mouse at 37°C).

### 5.8 Statistical analysis

Significant difference between the two species was determined by Student's *t* test separately for each of the three biologically relevant temperature contrasts that is, NMR at 30 vs. mice at 37°C, both species at 37°C, and both species at 30°C. Homogeneity of variance was assessed with Brown–Forsyth's when *n* > 5, or as a plot of residual when *n* < 5. In the absence of homoscedasticity, the Wilcoxon test was used to distinguish between the two species. Analyses were conducted using JMP 13.0 (SAS Institute, Cary, NC). In all cases, *p* < 0.05 was considered significant, sample size is presented in figure legends. Data are presented as mean ± *SEM*.

## CONFLICT OF INTEREST

None declared.

## AUTHOR CONTRIBUTION

D.M. was responsible for conducting the experiment and analyzing data. C.B. was responsible for acquiring consumption data with heart mitochondria. D.M and J.R.T. designed the experiments and wrote the manuscript in collaboration with M.E.P.
