# Supplementary figures and images for: The exceptional longevity of the naked mole‐rat may be explained by mitochondrial antioxidant defenses
Source: Aging Cell. 2019 Feb 15;18(3):e12916. doi: 10.1111/acel.12916 (PMC6516170; doi:10.1111/acel.12916)

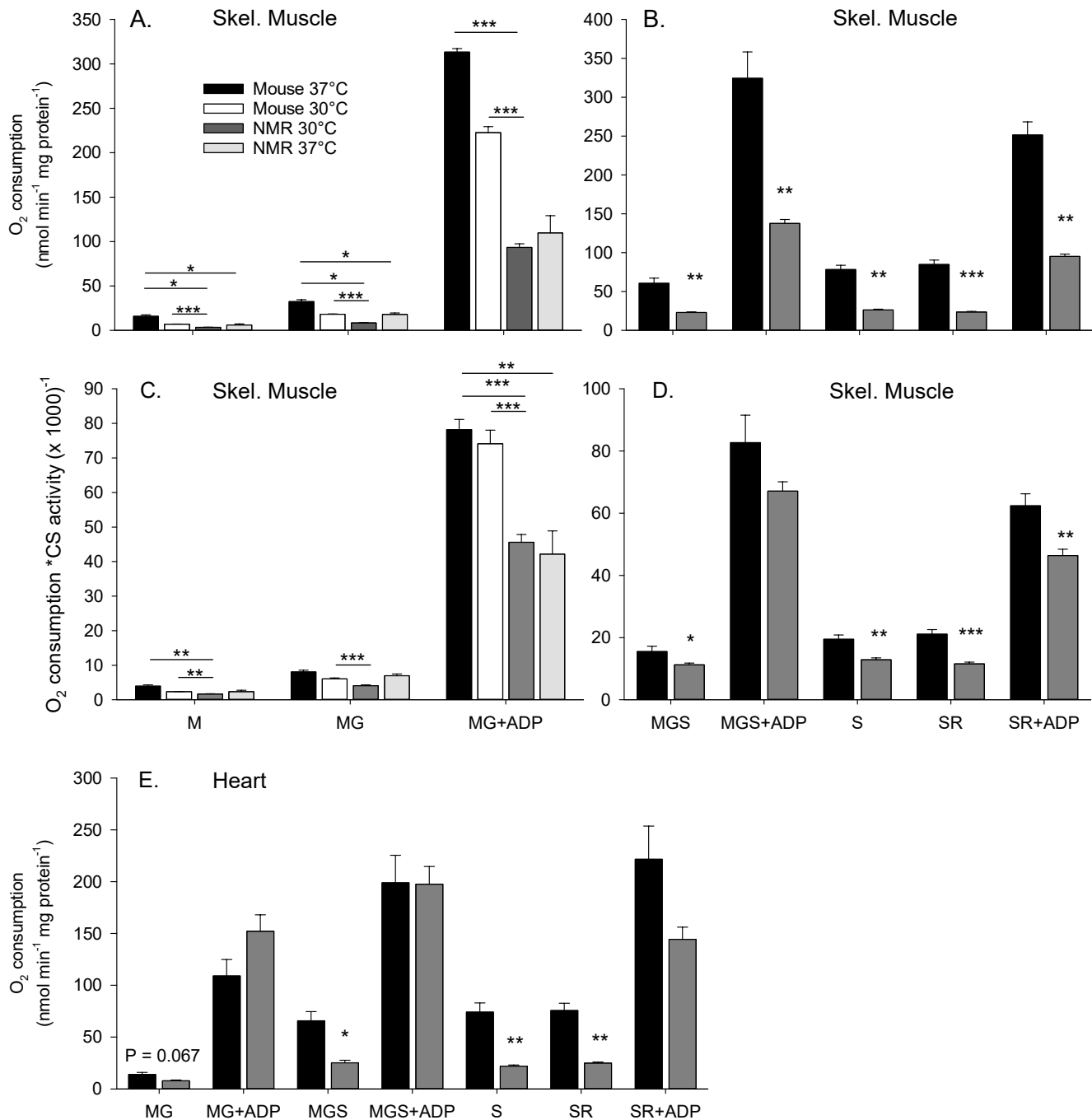

Supplement: Supplementary file 1 [file ACEL-18-e12916-s001.PDF]

RCR

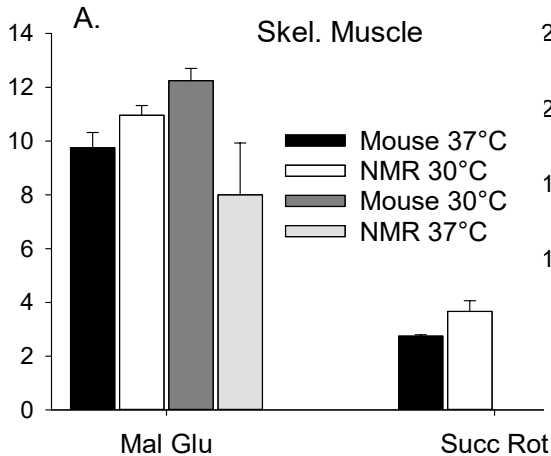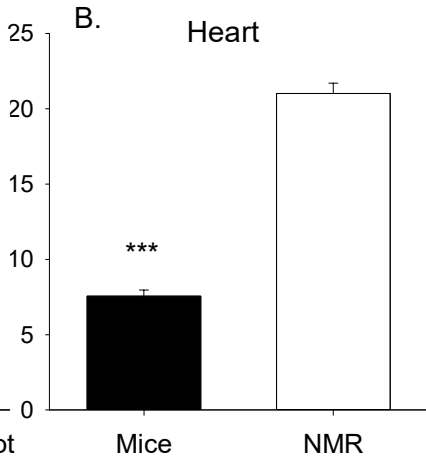

Supplement: Supplementary file 2 [file ACEL-18-e12916-s002.PDF]
