# Supplementary material for: The exceptional longevity of the naked mole‐rat may be explained by mitochondrial antioxidant defenses
Source: Aging Cell. 2019 Feb 15;18(3):e12916. doi: 10.1111/acel.12916 (PMC6516170; doi:10.1111/acel.12916)
Supplement: Supplementary file 3 [file ACEL-18-e12916-s003.docx]

## Supporting information

**Figure S1**. Oxygen consumption of isolated skeletal muscle (A-D) and heart (E) mitochondria. Data were acquired simultaneously with H_2_O_2_ consumption assays in CDNB pre-treated mitochondria in the presence of auranofin. A and C, for both species, assays were carried out at the species’ normal body temperature as well as that of the other species. Normal body temperatures are 30°C for the naked mole-rat (NMR), and 37°C for the mouse. B, D and E, assays were conducted at the species’ normal body temperature only. M = malate, G = glutamate, S = succinate, R = rotenone. Significant differences between species are t-tests with * for P < 0.05, ** for P < 0.01, and *** for P < 0.0001. Data are mean ± SEM. For mouse skeletal muscle n = 9 for MGS and MGS + ADP, n = 7 for all conditions with succinate, and n = 4 for others. For NMR skeletal muscle n = 6 for all assays at 30°C, and n = 2 for assays at 37°C. For heart, n = 5 for mice and 3 for NMR (four individuals pooled for n = 1).

**Figure S2**. Respiratory control ratios of skeletal muscle (A) and heart mitochondria (B). Data were measured simultaneously to H_2_O_2_ production assays with CDNB pre-treated mitochondria in the presence of auranofin. Mal Glu = malate + glutamate, Succ Rot = succinate + rotenone.
